# Ancient origin of somatic and visceral neurons

**DOI:** 10.1186/1741-7007-11-53

**Published:** 2013-04-30

**Authors:** Marc Nomaksteinsky, Stefan Kassabov, Zoubida Chettouh, Henri-Corto Stoeklé, Laure Bonnaud, Gilles Fortin, Eric R Kandel, Jean-François Brunet

**Affiliations:** 1 Institut de Biologie de l’École normale supérieure (IBENS), CNRS UMR8197, INSERM U1024, Paris, France; 2 Paris Sciences et Lettres University, Paris, France; 3 Department of Neuroscience, College of Physicians and Surgeons of Columbia University, New York, USA; 4 Museum National d’Histoire Naturelle, CNRS UMR7208, Université Pierre et Marie Curie, Paris, France; 5 Institut de Neurobiologie Alfred Fessard, CNRS UPR3294, Gif-sur-Yvette, 91198, France; 6 Howard Hughes Medical Institute, College of Physicians and Surgeons of Columbia University, New York, USA; 7 Kavli Institute for Brain Science, College of Physicians and Surgeons of Columbia University, New York, USA

**Keywords:** Sensory neurons, Motor neurons, Evolution, Transcription factors, Mollusks, Lophotrochozoa, Lymnaea, Aplysia, Sepia, Phox2, Brn3, Mnx

## Abstract

**Background:**

A key to understanding the evolution of the nervous system on a large phylogenetic scale is the identification of homologous neuronal types. Here, we focus this search on the sensory and motor neurons of bilaterians, exploiting their well-defined molecular signatures in vertebrates. Sensorimotor circuits in vertebrates are of two types: somatic (that sense the environment and respond by shaping bodily motions) and visceral (that sense the interior milieu and respond by regulating vital functions). These circuits differ by a small set of largely dedicated transcriptional determinants: *Brn3* is expressed in many somatic sensory neurons, first and second order (among which mechanoreceptors are uniquely marked by the *Brn3+/Islet1+/Drgx+* signature), somatic motoneurons uniquely co-express *Lhx3/4* and *Mnx1*, while the vast majority of neurons, sensory and motor, involved in respiration, blood circulation or digestion are molecularly defined by their expression and dependence on the pan-visceral determinant *Phox2b*.

**Results:**

We explore the status of the sensorimotor transcriptional code of vertebrates in mollusks, a lophotrochozoa clade that provides a rich repertoire of physiologically identified neurons. In the gastropods *Lymnaea stagnalis* and *Aplysia californica*, we show that homologues of *Brn3, Drgx, Islet1, Mnx1, Lhx3/4* and *Phox2b* differentially mark neurons with mechanoreceptive, locomotory and cardiorespiratory functions. Moreover, in the cephalopod *Sepia officinalis*, we show that *Phox2* marks the stellate ganglion (in line with the respiratory — that is, visceral— ancestral role of the mantle, its target organ), while the anterior pedal ganglion, which controls the prehensile and locomotory arms, expresses *Mnx*.

**Conclusions:**

Despite considerable divergence in overall neural architecture, a molecular underpinning for the functional allocation of neurons to interactions with the environment or to homeostasis was inherited from the urbilaterian ancestor by contemporary protostomes and deuterostomes.

## Background

For several decades, molecular data have been used to define homologous regions in the nervous system of distant phyla. More recently, homology search has moved to the cell level, using conserved neuronal-type specific molecular signatures [1-5] (and reference [6] for review). This approach provides a novel window on the complexity of ancestral nervous systems, and sets the stage, with unprecedented detail, for an understanding of what has changed or been conserved during their large-scale evolution. In this paper, we undertake a comparison of sensorimotor circuits across the protostome/deuterostome boundary, which is, thus, informative about the nervous system of Urbilateria.

**Figure 1:**
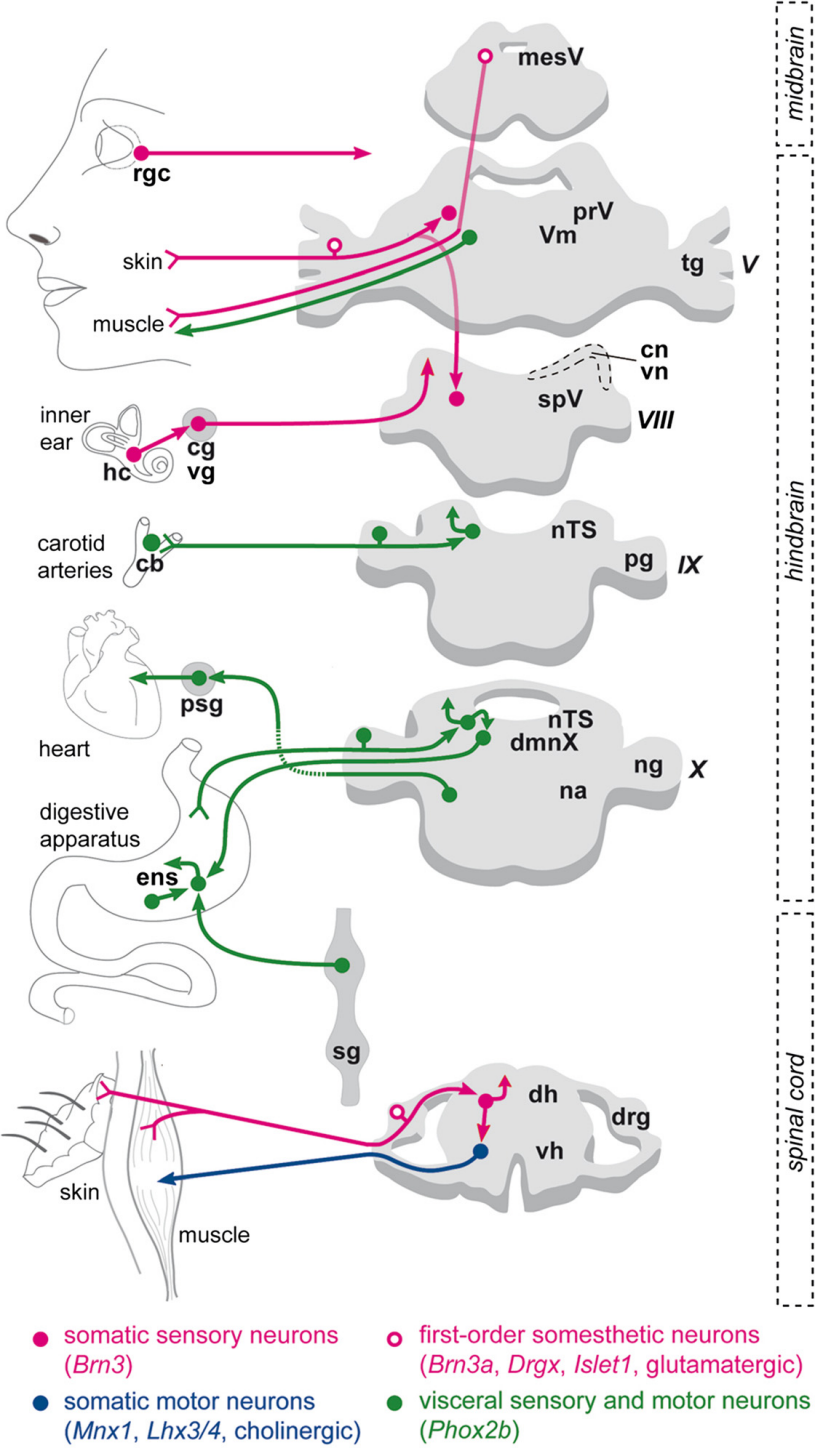
**Somatic and visceral neurons are distinguished by a small set of transcription factors in vertebrates.** Schematic of the main neuronal types that form the sensorimotor circuits in vertebrates, and their molecular code. An exemplar of each category is shown with its target organ. Magenta: somatic sensory neurons (with a history of *Brn3* expression), including somesthetic neurons (mechanoreceptors, proprioceptors, thermoreceptors and nociceptors) of the trigeminal and dorsal root ganglia, and at least some of their post-synaptic partners in the nuclei of the trigeminal nerve and dorsal horn of the spinal cord, respectively, retinal ganglionic cells and vestibulo-cochlear neurons, and the latters’ targets, the hair cells of the inner ear. Blue: somatic motoneurons (with a history of *Mnx1* and *Lhx3/4* expression) that control bodily motions. Green: neurons of the visceral sensory pathways and motor outflow (with a history of *Phox2b* expression): first-order sensory neurons in epibranchial ganglia and second-order sensory neurons of the nTS; the carotid body; sympathetic, parasympathetic and enteric ganglionic neurons, and preganglionic neurons to the latter two; and branchiomotor neurons (respiratory in aquatic vertebrates). V, trigeminal nerve; Vm, motor nucleus of the trigeminal nerve; VIII, vestibulo-cochlear nerve; IX, glossopharyngeal nerve; X, vagal nerve; cb, carotid body; cg, cochlear ganglion; cn, cochlear nuclei; dh, dorsal horn; dmnX, dorsal motor nucleus of the vagus nerve; drg, dorsal root ganglion; hc, cochlear and vestibular hair cells; ens, enteric nervous system; mesV, mesencephalic nucleus of the trigeminal nerve; na, nucleus ambiguus; ng, nodose ganglion; nTS, nucleus of the solitary tract; pg, petrosal ganglion; prV, principal nucleus of the trigeminal nerve; psg, parasympathetic ganglion; rgc, retinal ganglion cells; sg, sympathetic ganglia; spV, spinal nucleus of the trigeminal nerve; tg, trigeminal ganglion; vg, vestibular ganglion; vh, ventral horn; vn, vestibular nuclei.

Ever since Bichat distinguished the ‘organic’ and ‘animal’ lives [7], the vertebrate body has been construed as a dual entity, one part somatic (engaged with the outside world), the other visceral (concerned with bodily homeostasis). Paralleling this distinction, the sensorimotor circuits passing through the spinal cord and brainstem are divided into somatic and visceral. Somatic circuits are responsible for the somesthetic, visual or auditory perception of the environment and locomotory responses. The visceral circuits are responsible for sensing parameters of the interior milieu, such as arterial pressure, blood gases and various chemosensory modalities including taste, and feedback regulation of the cardiovascular, respiratory and digestive organs. Specific anatomic features and embryonic origins have been progressively discovered for these circuits. For example, somatic and visceral neurons in the vertebrate hindbrain are born and settle at distinct dorso-ventral levels [8]. In the peripheral nervous system, first-order visceral sensory neurons emerge from epibranchial placodes, whereas somatic ones derive from dorso-lateral placodes or the neural crest [9]. In his landmark monograph on the subject, A.S. Romer synthesized a century of observations on the ‘duality’ of the vertebrates, made up of two bodies (and nervous systems), one visceral and one somatic, ‘imperfectly welded’ on each other [10]. In the past years, after decades of neglect, the proposed dichotomy of the vertebrate nervous system has found, piecemeal, an unexpected molecular basis, in the form of transcription factors that globally distinguish somatic from visceral neurons (Figure 1) and are largely, if not completely, restricted to them. Most somatic sensory neurons, first- and second-order, express the POU domain genes *Brn3*: the touch and pain receptors of the dorsal root and cranial ganglia, auditory and vestibular neurons, ganglionic cells of the retina, and many relay sensory neurons of the dorsal horn of the spinal cord —even if, in the latter, expression is transient and not entirely charted [11,12] (and references therein). *Brn3* is required in all the peripheral cell types, its role in the central nervous system (CNS) being yet unexplored. Somatic motoneurons express and require the combination of the homeobox genes *Mnx1*[13] and *Lhx3/4*[14]. Finally, the vast majority of visceral neurons, sensory and motor, express and require the homeobox gene *Phox2b:* visceral sensory ganglia and their target, the nucleus of the solitary tract, all autonomic ganglia; visceral motoneurons of the medulla; branchial motoneurons (respiratory —that is, visceral— in the ancestral vertebrates) and chemosensory structures, such as the carotid body and the retrotrapezoid nucleus [12,15-17]. This simple, non-combinatorial code provides new tools to probe the ancestry of these broad neuronal categories.

For this study, we turned to mollusks, for two reasons: first, they belong to the phylum Lophotrochozoa which, genetically less derived than Ecdysozoa, is particularly valuable for comparisons across Bilateria [18]; and second, mollusks have been studied by neurophysiologists for decades and consequently provide a unique catalogue of identified neurons with somatic or visceral functions [19] that allow rigorous tests of association with specific molecular signatures. Using as model systems two gastropods —the opisthobranch *Aplysia californica* and the pulmonate *Lymnaea stagnalis*— and the decapodiform cephalopod *Sepia officinalis*, we show that physiologically defined somatic motor and sensory neurons and visceral motoneurons share, respectively, the *Mnx/Lhx3/4*, *Brn3* and *Phox2* transcriptional signature of their vertebrate counterparts, and we discuss the evolutionary implications of this conservation across Bilateria.

## Results

### Shared molecular signature of gastropod and vertebrate mechanosensory neurons

**Figure 2:**
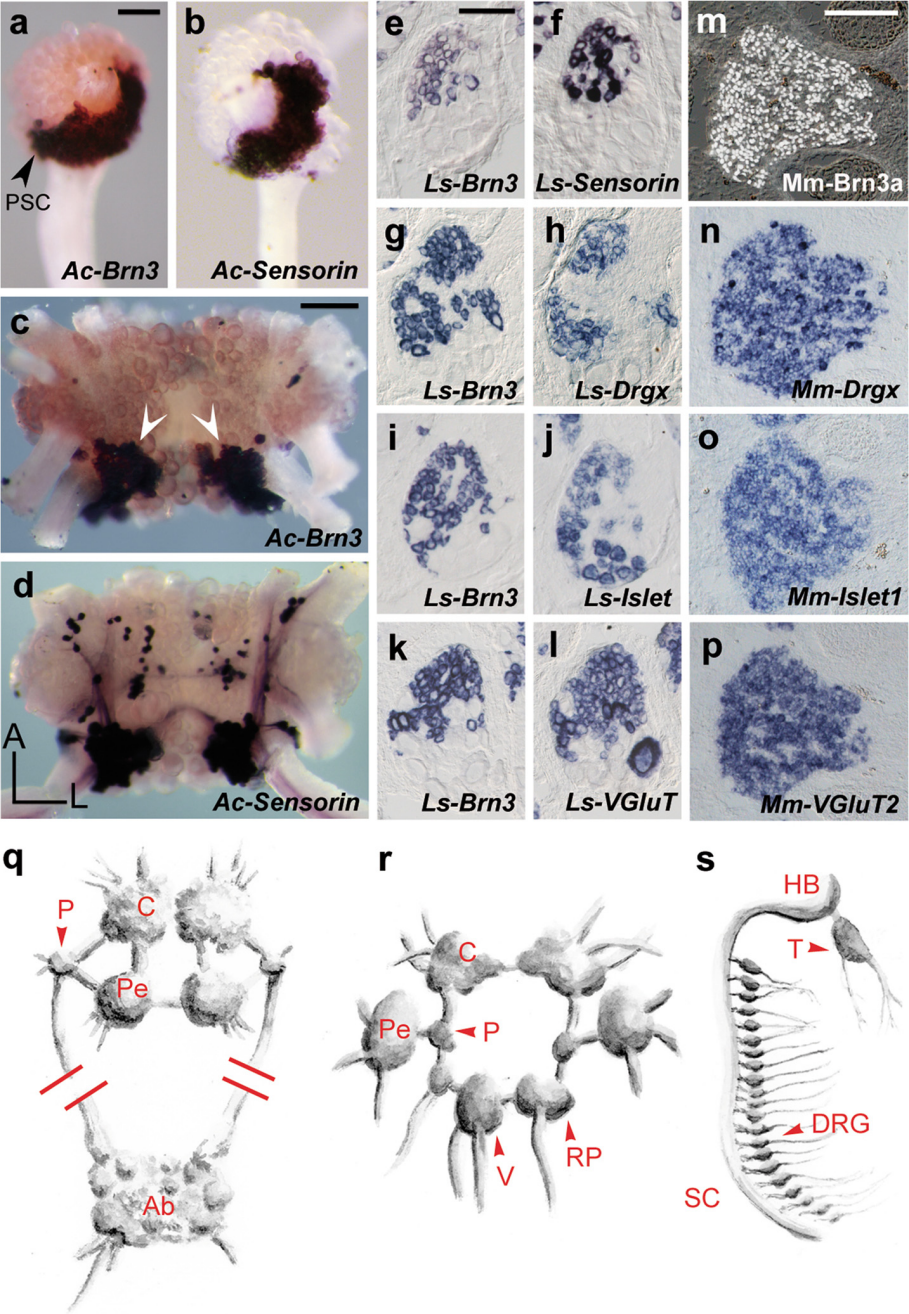
**Transcriptional code of mechanoreceptors in gastropods and vertebrates.** (**a**-**d**) Whole-mounts of the pleural (**a**,**b**) or cerebral ganglion (**c**,**d**) (ventral view) of *Aplysia californica* (*Ac*) hybridized with the indicated probes. In the cerebral ganglia, *Brn3* is expressed in the *Sensorin*^*+*^ J and K sensory clusters (white arrowheads) [23] but not in the scattered *Sensorin*^*+*^ cells. Projections from the sensory clusters contain the *Sensorin* mRNA and are detected by *in situ* hybridization [23]. (**e**-**l**) Consecutive sagittal sections of the pleural ganglion of *Lymnaea stagnalis* (*Ls*) showing coexpression of the indicated genes. (**m**-**p**) Consecutive sagittal sections of a dorsal root ganglion in *Mus musculus* (*Mm*) showing coexpression of the indicated genes. In *Lymnaea* pleural ganglia, *Islet* and *VGluT* are also expressed in large *Brn3*^*—*^ neurons at the ventral pole. (**q**-**s**): schematic of the nervous system of *Aplysia californica* (**q**), *Lymnaea stagnalis* (**r**) and *Mus musculus* (**s**). A, anterior; Ab, abdominal ganglion; C, cerebral ganglion; DRG, dorsal root ganglion; HB, hindbrain; L, left; P, pleural ganglion; Pe, pedal ganglion; PSC, pleural sensory cluster; RP, right parietal ganglion; SC, spinal cord; T, trigeminal ganglion; V, visceral ganglion. Scale bars, 100 μm (a,b); 200 μm (c,d); 100 μm (e,g,i,k); 100 μm (f,h,j,l); 200 μm (m-p).

In vertebrates, the vast majority of sensory neurons that perceive the environment (with the exception of olfactory neurons) express paralogues of the *POU-IV/Brn3* homeogene family, henceforth collectively called *Brn3*. These include mechanoreceptors, proprioceptors, thermoreceptors and nociceptors of the dorsal root and trigeminal ganglia, retinal ganglion cells, vestibular and cochlear sensory neurons and inner hair cells [11] (and references therein), as well as many second-order neurons in these somatic sensory pathways [12] (and references therein). The evolutionary stability of this genetic signature across Bilateria has been uncertain so far, based on the study of model ecdysozoans: mechanoreceptors have not been reported to express the orthologue of *Brn3* in *Drosophila*, where it labels olfactory neurons instead [20], and they form only a small fraction of the 57 neurons that express the *Brn3* orthologue in *Caenorhabditiselegans*[21]. In the gastropod *Haliotis asinina*, expression of *Brn3* in patches of larval ectoderm has been interpreted as marking peripheral sensory structures [22]. We examined the case of *A. californica*, where mechanoreception and nociception are mediated, at least in part, by clusters of small-size sensory neurons in several ganglia of the CNS, which synthesize the neuropeptide sensorin A [23]. We cloned the *Aplysia* orthologue of *Brn3* [see Additional file 1: Figure S1a] and found it expressed in the *Sensorin*^*+*^ neuronal clusters and restricted to them in the CNS (Figure 2a-d,q for the pleural and cerebral clusters). In another gastropod, the pulmonate *L. stagnalis*, similar clusters of small *Brn3*^*+*^ neurons occurred in several ganglia (Figure 2e for the pleural ganglion), many of which expressed the *Lymnaea* orthologue of *Sensorin* (Figure 2f and Additional file 1: Figure S2) and are thus most likely homologous to the mechanoreceptors of *Aplysia*. To further elucidate the molecular signature of these cells, we cloned the *Lymnaea* orthologues of *Drgx* [see Additional file 1: Figure S1b], largely restricted to sensory neurons in vertebrates [24], and of *Islet1* [see Additional file 1: Figure S1c], expressed in all vertebrate sensory neurons [25] (and motoneurons). Both were coexpressed with *Brn3* in *Lymnaea* (Figure 2g-j,r) as is the case for their orthologue in the touch and pain receptors of the mammalian dorsal root ganglia (Figure 2m-o,s). In addition, *Lymnaea* sensory clusters, like their vertebrate counterparts, expressed the vesicular glutamate transporter *VGluT* (Figure 2k,l,p), in line with the glutamatergic phenotype of molluscan mechanoreceptors [26]. Thus, the unique *Brn3*^*+*^/*Drgx*^*+*^/*Islet*^*+*^*/VGluT*^*+*^ transcriptional signature of first-order somatic sensory neurons in vertebrates, is also a hallmark of their molluscan counterparts.

### Shared molecular distinction between somatic and visceral motoneurons in gastropods, cephalopods and vertebrates

**Figure 3:**
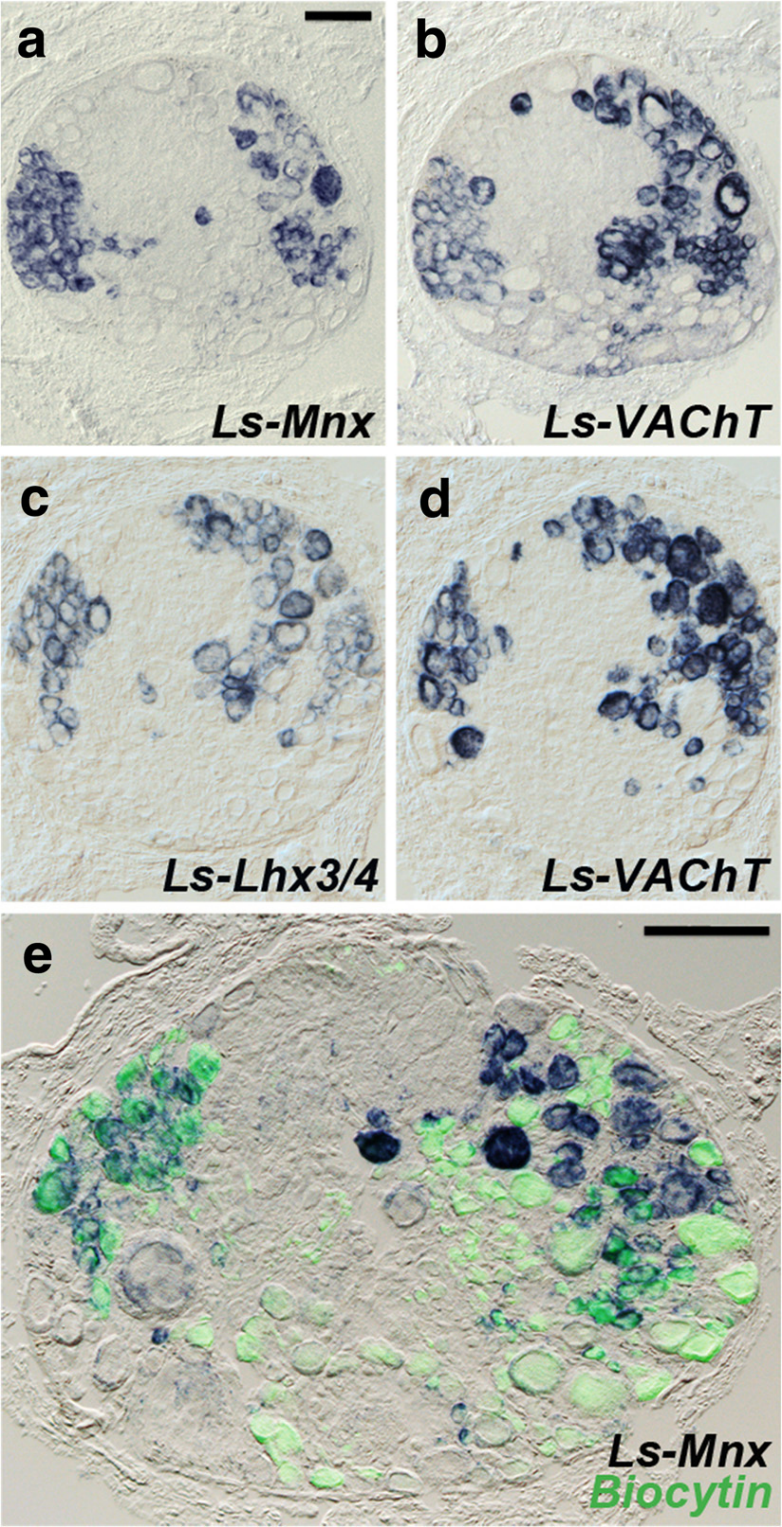
**Transcriptional code of putative somatic motoneurons in the pedal ganglia of *Lymnaea stagnalis*.** Consecutive sagittal sections (respectively **a**,**b** and **c**,**d**) through the pedal ganglion hybridized with the indicated probes. In **e**, the ganglion was hybridized with *Ls-Mnx* after retrograde filling with biocytin through the three pedal nerves to the foot. The three nerves to the neck and columella, also involved in locomotion, were not filled. The anterior cluster of Mnx^+^ cells is filled, as well as about half of the posterior cluster. Scale bars, 100 μm.

In vertebrates, locomotion depends on somitic muscles, innervated by spinal cord motoneurons that depend on the homeogenes *Mnx1*[13] and *Lhx3/4*[14] and use acetylcholine as neurotransmitter. In gastropods, the muscles of locomotion (in the foot and body wall or attached to the columella) are innervated by the pedal ganglia [27]. We cloned *Ls-Mnx*, the *Lymnaea* orthologue of *Mnx1* [see Additional file 1: Figure S1d] and found that it was largely restricted to two clusters of neurons in the pedal ganglia (Figure 3a), which coexpressed the orthologues of *Lhx3/4* (Figure 3c and Additional file 1: Figure S1e) and the vesicular acetylcholine transporter *VAChT* (Figure 3b,d). At least some of these cells projected in the pedal nerves, as assessed by retrograde filling (Figure 3e-g) and most likely are locomotory neurons. These data extend the observations that some motoneurons in *Drosophila* also express *Lim3* (the *Lhx3/4* orthologue) and *Mnx*[28,29], that some *C. elegans* motoneurons express a *Mnx* orthologue [30], and that the *Nkx6*^*+*^ domain of the annelid nerve cord gives rise to *Mnx*^*+*^/*VAChT*^*+*^ putative motoneurons [31]. Thus, the *Mnx*^*+*^*/Lhx3/4*^*+*^ molecular signature has been associated with somatic motoneurons since the origin of bilaterians.

**Figure 4:**
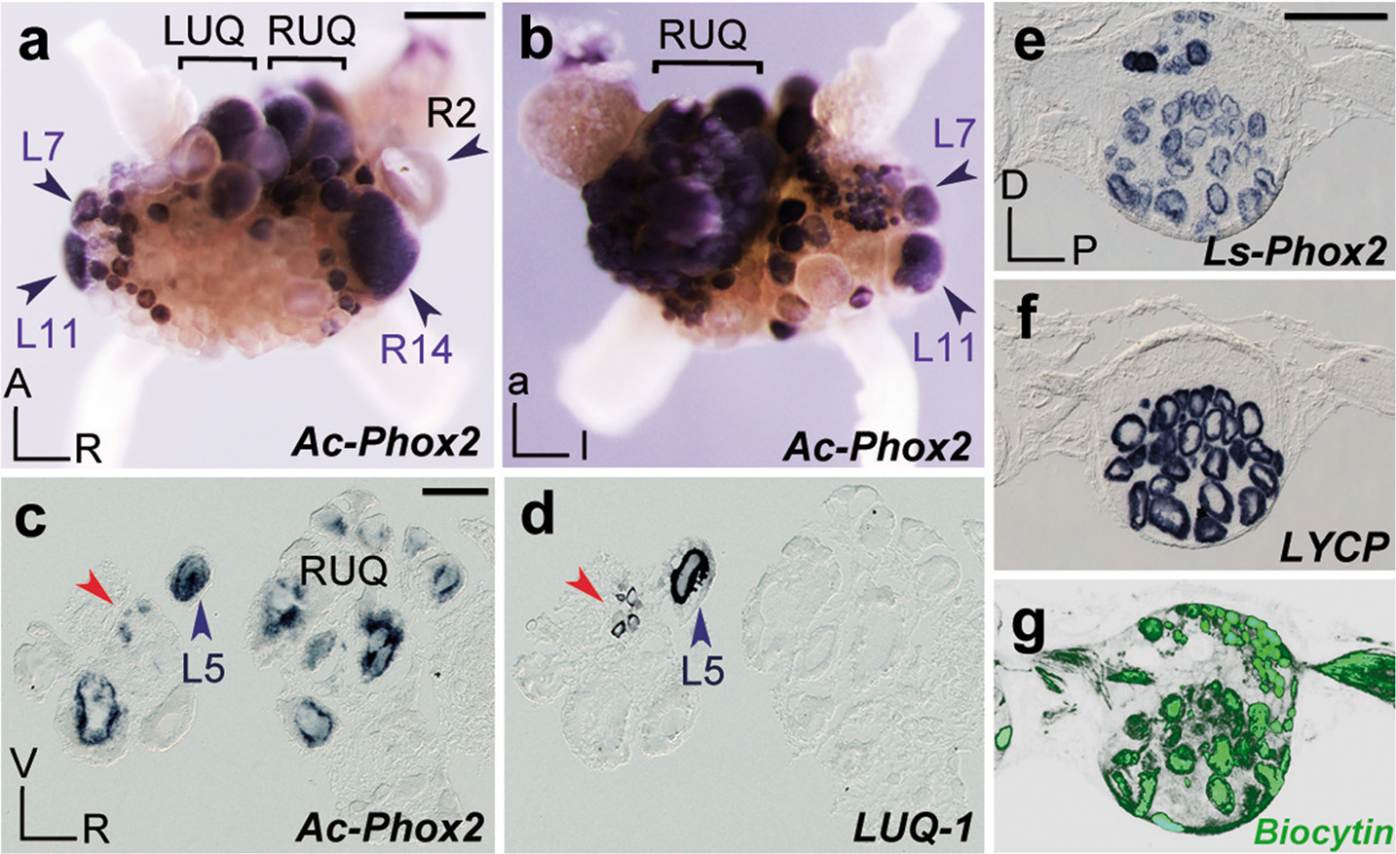
**Expression of *Phox2 *in identified visceral motoneurons in gastropods.** (**a**-**d**) Abdominal ganglion of *A. californica* hybridized on whole-mounts (**a**: dorsal view, **b**: ventral view) or transverse sections (**c**,**d**) with the indicated probes. In **a**, the giant R2 neuron is unlabeled. Red arrowheads in **c**,**d**: small neurons co-expressing *Phox2* and *LUQ-1*. (**e**-**g**) Consecutive sagittal sections of the right parietal ganglion of *L. stagnalis* hybridized with the indicated probes, after it was filled with biocytin through the internal and external right parietal nerves (**g**). A, anterior; D, dorsal; L, left; P, posterior; LUQ, left upper quadrant; R, right; *LYCP*, LYC pro-peptide; RUQ: right upper quadrant; V, ventral. Scale bars, 100 μm (**a**,**b**); 100 μm (**c**,**d**); 200 μm (**e**-**g**).

We next examined the visceral nervous system, whose sensorimotor circuits in vertebrates largely coincide with the expression pattern of the paralogous homeogenes *Phox2a* and *Phox2b* (hereafter collectively designated as *Phox2*) and depend on the latter for their formation [12,15-17]. Among deuterostomes, we previously found that the *Ciona intestinalis* orthologue of *Phox2* is specifically expressed in neurons of the cerebral ganglion of postmetamorphic animals that motorize the respiratory and digestive ‘branchial basket’ [2]. Concerning protostomes, the *C. elegans* orthologue of *Phox2* marks specifically five neurons [32] whose function is either unknown (the SIAs) or hard to classify as somatic or visceral (ALA, involved in behavioral quiescence [33]). The inconclusiveness of the latter finding is compounded by the fact that most visceral organs in *C. elegans* either lack innervation (such as the intestine) or are missing altogether (such as a cardiovascular or a respiratory apparatus). In *A. californica*, several neurons with cardiovascular, respiratory or excretory functions have been identified by size and location in the abdominal ganglion. One of the best documented, the multimodal motoneuron L7, directly innervates the muscles of the gill, siphon, epineural sheath, heart and abdominal aorta [34] (and references therein), and serves as premotor neuron for the branchial ganglion [35]. We cloned *Ac-Phox2*, the *Aplysia* orthologue of *Phox2* [see Additional file 1: Figure S1b] and found it expressed in L7, recognizable by its large size and position at the left border of the abdominal ganglion, rostral to the other large (and also *Phox2*^*+*^) neuron in the region, L11, which projects in the genital nerve but whose function is unknown (Figures 4a,b and 2q). Among the other neurons expressing *Phox2* was a large cluster occupying the ‘right upper quadrant’ of the ganglion (Figure 4a,b), which includes the giant R3-R13 cells [36] that innervate the heart, major arteries and veins, digestive gland sheath and kidney [37]. Many neurons that are smaller than the R3-R13 cells were also positive for *Phox2* in this region. These might correspond to the extra cells found to share with R3-R13 electrical activity [38], immunoreactivity for the cardiomodulator peptide NdWFamide [39] and, thus, possibly a function. A giant *Phox2*^+^ cell located close to the branchial nerve corresponded to R14 (Figure 4a), which shares its targets with the R3-R13 group [37]. The R3-R14 neurons are thought to modulate heart beat frequency and local aspects of circulatory physiology using, among others, the peptide HRBP [40] as neurotransmitter. The kidney also receives input from giant neurons of the ‘left upper quadrant’. Among those, L5 is uniquely identified by expression of the peptide encoding the *LUQ-1* gene [41], allowing us to show on consecutive sections that it was *Phox2*^+^ (Figure 4c,d). In *Lymnaea*, large ‘Light Yellow Cells’ (LYCs) [42] are considered homologous to the R3-R14 neurons on the basis of their size, color, location, synthesis of a peptide similar to HRBP [43] and projections to the heart, aorta, kidney, and connective tissue of the CNS [44]. We cloned *Lymnaea Phox2* [see Additional file 1: Figure S1b] and found that it was expressed in all LYCs of the ventro-lateral lobe of the right parietal ganglion, identified by their position and expression of the *LYC* pro-peptide (Figures 4e,f and 2r). Retrograde filling showed that they projected in the right parietal nerves (Figure 4g), which innervate the mantle cavity and pneumostome [45], suggesting an additional role in ventilation.

**Figure 5:**
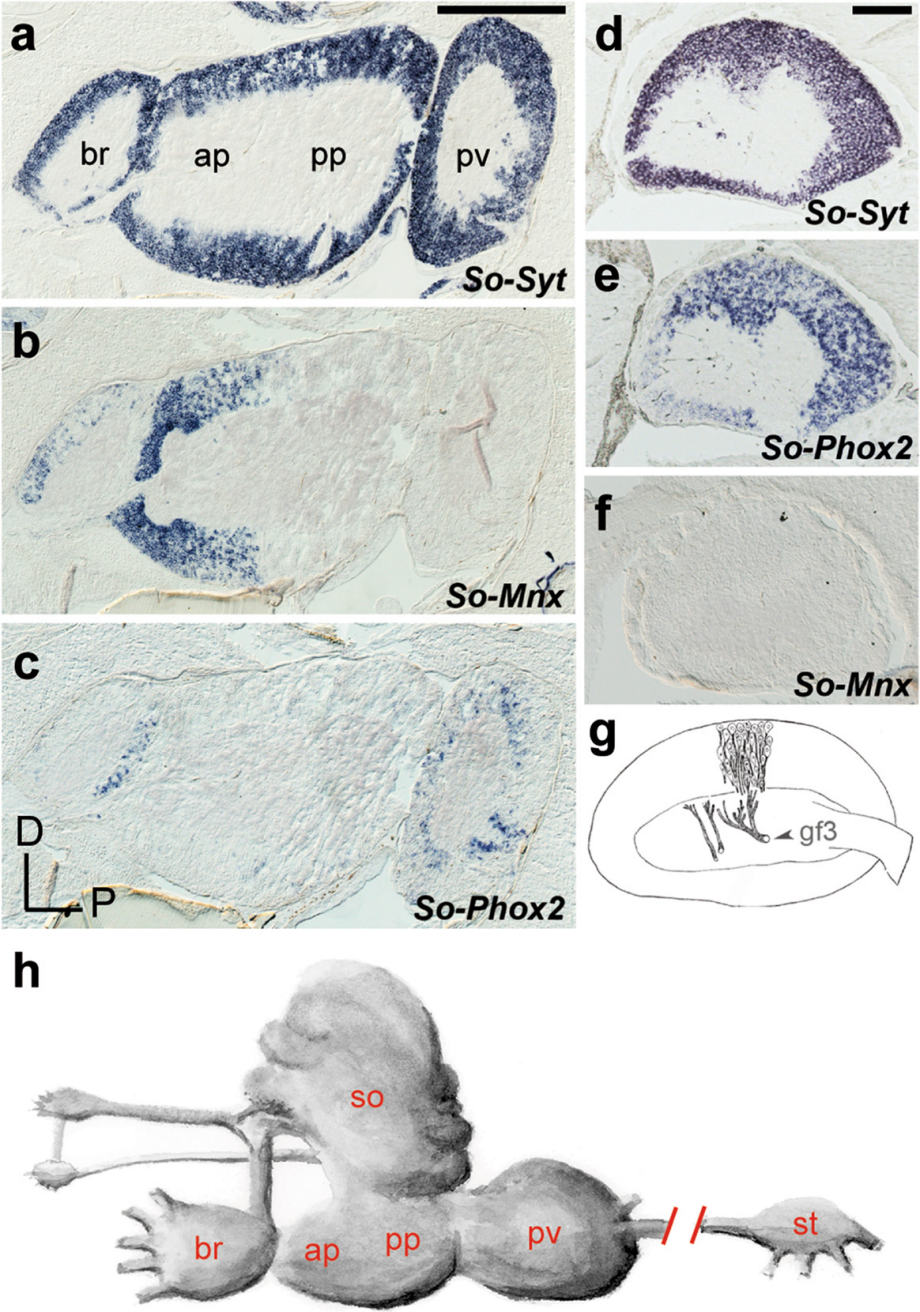
**Expression of *Phox2 *and *Mnx *in the subesophageal and stellate ganglia of *Sepia officinalis*.** (**a**-**f**) Consecutive sagittal sections through the subesophageal ganglonic mass (**a**-**c**) or a stellate ganglion (**d**-**f**) hybridized with the indicated probes. *Synaptotagmin 1/2/5* (*So-Syt*) is used as a pan-neuronal marker (**a**,**d** and Additional file 1: Figure S1f). Apart from the expressions described in the text, the brachial ganglion contains *So*-*Mnx*^*+*^ neurons, in line with its role in movement of the arms, but also *So-Phox2*^*+*^ neurons in its posterior wall, whose function has not been described so far. (**g**) Drawing, reproduced with permission from Figure thirteen of reference [49], showing the way in which the axons of stellate ganglion neurons progressively fuse to form the third order giant fibers (gf3) that allow jet-propulsion. (**h**) Schematic of the central nervous system and stellate ganglion of *Sepia*. br, brachial ganglion; D, dorsal; P, posterior; pa, anterior pedal ganglion; pp, posterior pedal ganglion; pv, palliovisceral ganglion; so, supraesophageal mass; st, stellate ganglion. Scale bars, 500 μm (**a**-**c**); 200 μm (**d**-**f**).

As a further test of the vertebrate-like molecular signature of visceral and somatic motoneurons in mollusks, we explored a second molluscan clade, Cephalopoda. In decapodiform cephalopods (squid and cuttlefish), the brachial ganglion and anterior part of the pedal ganglion contain motoneurons for the prehensile and locomotory arms, while the palliovisceral ganglion contains motoneurons for the visceral mass [46,47]. We cloned the orthologues of *Mnx* and *Phox2* in *S. officinalis* [See Additional file 1: Figure S1b,d] and found that *Mnx*, but not *Phox2*, was expressed throughout the anterior pedal lobe (Figure 5a-c) while *Phox2*, but not *Mnx,* was expressed in the palliovisceral ganglion (Figure 5a-c). Some of the palliovisceral ganglionic neurons are presynaptic to the motoneurons of the mantle, which make up most of the stellate ganglion, a synapomorphy of this clade. *Phox2*, but not *Mnx*, was expressed in most stellate ganglionic neurons (Figure 5d-f). Thus, mantle motoneurons, which control water flow over the gills, express *Phox2*, like the branchial motoneurons that perform the same function in fish [16,48]. Apart from their respiratory function, motoneurons to the mantle (whose axons fuse to form the so-called ‘giant nerve fibers’— reference 49 and Figure 5g) allow rapid water ejection and jet-propulsion of the animal. The molecular identity of these escape motoneurons thus correlates with the ancestral function of the target organ (respiratory, that is, visceral) rather than with its additional, derived function (locomotory, that is, somatic). A conceptually similar but inverse situation can be described in terrestrial (that is, air breathing) vertebrates, whose respiratory motoneurons (projecting to the intercostal, abdominal and diaphragmatic muscles) are somatic, that is, have kept the identity matching the embryonic origin (somitic) and ancestral function (locomotory, except for the diaphragm of mammals) of their target muscles.

Hence, molluscan motoneurons that innervate the viscera are distinguished from those that innervate the locomotory muscles by the same transcriptional code (respectively *Phox2* and *Mnx/Lhx3/4*) as their vertebrate counterparts.

## Discussion

We have shown that molecular signatures for neurons with somatic (that is, relational) versus visceral (that is, homeostatic) functions are conserved between vertebrates and mollusks. Visceral motoneurons, (such as cardiorespiratory neurons) express the orthologue of the vertebate pan-visceral determinant *Phox2b* in opisthobranch and pulmonate gastropods and a decapodiform cephalopod. No other transcription factor or neurotransmitter phenotype marks these neurons, specifically or exhaustively, in vertebrates, precluding a more complex signature. However, *Phox2b* expression is highly selective for visceral neurons (both motor and sensory) in vertebrates [16] and, thus, in combination with hodological criteria, constitutes a strong argument for homology. Neurons with modalities clearly equivalent to those of visceral sensory neurons in vertebrates (which monitor taste or blood pressure for example) are not described to our knowledge in mollusks, precluding exploration of this broad neuronal identity. Of note, in vertebrates, the viscerosensory phenotype is imposed by *Phox2b* on a somatosensory default identity [12], suggesting that the former is evolutionarily more recent than the latter. Locomotory (somatic) motor neurons express the orthologues of the homeobox genes *Mnx1* and *Lhx3/4* and *VAChT* in both gastropods and cephalopods, like their counterparts in the spinal cord of vertebrates. Finally, somatic sensory neurons (such as mechanoreceptors), characterized in gastropods by previous electrophysiological studies or expression of the peptide sensorin A, selectively express the orthologues of the homeodomain genes *Brn3*, *Drgx*, and *Islet1* and *VGluT*, like their physiological counterparts in the dorsal root and trigeminal ganglia of vertebrates.

What evolutionary relationship can explain the conservation of molecular signatures in neurons with visceral versus somatic functions between deuterostomes and protostomes? The simplest hypothesis is that the cells are phylogenetically homologous, that is, one could trace their ancestry to an original neuron or neuronal cluster in the common ancestor, as was proposed for ciliary photoreceptors in annelids and vertebrates [1] or for branchial motoneurons in vertebrates and urochordates [2]. This might also be the case for somatic motoneurons in vertebrates and lophotrochozoans: their distribution is spatially discrete in each phylum and reconcilable between phyla. In vertebrates, locomotory *Mnx*^*+*^ neurons are born in a ventral *Nkx6.2*^*+*^ domain of the spinal cord, topologically and molecularly similar to the ventral domain of the nerve cord of annelids, which produces *Mnx*^*+*^*/VAChT*^*+*^ neurons, presumably motor [31]. In mollusks, they are restricted to the pedal ganglia, conceivably homologous to the ventral nerve cord of annelids.

On the other hand, phylogenetic homology between vertebrates and mollusks is unlikely, at least in most cases, for somatic sensory neurons and visceral motor neurons, due to their anatomical distribution, widespread in each species (Figures 1, 3, 4) and hard to reconcile between them. Moreover, *Phox2* is expressed in synapomorphic structures of vertebrates (the neural crest-derived autonomic ganglia) and cephalopods (the stellate ganglion). In this case, the most likely evolutionary scenario is that, in the last common ancestor, a ‘seminal regulatory interaction’ [50] arose between *Brn3* and the somatic sensory phenotype and between *Phox2* and the visceral phenotype. Although no target gene has been uncovered yet that would explain the physiological dichotomy these transcription factors specify, one can hypothesize, for example, that they direct axonal projections towards somatic versus visceral targets. Subsequently, this regulatory interaction would have been conserved, while *Brn3* and *Phox2* acquired additional expression sites, giving rise to novel groups of cells of the same broad type along each evolutionary lineage. According to this view, the relationship between the different kinds of somatosensory neurons or visceromotor neurons would be neither of phylogenetic homology (between species) nor of ‘sister cell types’ (within a species [6]), but instead, both between and within species, of ‘deep’ [51,52] or ‘generative’ [53] homology, akin to that proposed for bilaterian appendages.

## Conclusions

Regardless of the exact nature of what has been conserved across the protostome-deuterostome boundary, either neuronal groups or regulatory links between transcription factors and neuronal traits, our data show that the viscerosomatic duality of the nervous system, as described in vertebrates, was already part of the urbilaterian body plan. Some of its components might be even more ancient, as suggested by expression of a *Brn3* orthologue in the sensory ‘rhopalia’ of scyphozoan and cubozoan jellyfish [54]. To our knowledge, *Mnx* has not been analyzed in the nervous system of cnidarians. Finally, no straightforward orthologue of *Phox2* emerges from sequence analysis of paired-like homeobox genes in Cnidaria [see Additional file 1: Figure S1b], which complicates the search for a pre-bilaterian origin of the visceral nervous system.

## Methods

### Tissue processing

Ganglia from adult *A. californica* (100 to 150 g) were dissected in artificial seawater (460 mM NaCl, 10 mM KCl, 55 mM MgCl_2_, 11 mM, CaCl_2_, 10 mM (4-(2-hydroxyethyl)-1-piperazineethanesulfonic acid (HEPES), pH 7.6), protease digested for two hours at 37°C, fixed with 4% paraformaldehyde (4% PFA) in phosphate buffered saline (PBS) overnight at 4°C, desheathed and dehydrated in ethanol. *S. officinalis* fertilized eggs were removed from their envelopes in artificial seawater and fixed overnight with 4% PFA when they reached stage 29 [55], dechorionated, fixed again with 4% PFA for a whole day and dehydrated in methanol. Samples destined to be sectioned were rehydrated in PBST (PBS, 0.1% Tween-20), incubated overnight at 4°C in (PBS, 15% sucrose), incubated for 50 minutes at 65°C in gelatine (PBS, 7.5% gelatine, 15% sucrose, pH 7.2), embedded in gelatine, frozen for one minute at −50°C in isopentane and kept at −80°C until sectioning. Ganglia from adult *L. stagnalis* (20 to 40 mm) specimens were dissected in a physiological solution (40 mM NaCl, 1.7 mM KCl, 1.5 mM MgCl_2_, 4.1 mM CaCl_2_, 10 mM HEPES, pH7.9), fixed with 4% PFA and processed for gelatine embedding as described above. cDNA from *Rattus norvegicus Drgx*, *R. norvegicus Islet1* and *R. norvegicus VGluT2* sequences and a mouse anti-Brn3a monoclonal antibody (MAB1585 Millipore, Billerica, MA, USA) were used for mice procedures.

### *In situ* hybridization on sections

Frozen sections (12 μm) were thawed and air dried, washed briefly in PBST, treated 2 × 10 minutes with RIPA buffer (150 mM NaCl, 1% NP-40, 0.5% Na deoxycholate, 0.1% SDS, 1 mM ethylenediaminetetraacetic acid (EDTA), 50 mM Tris pH 8.0), postfixed with 4% PFA for 15 minutes, washed 3 × 5 minutes in PBST, acetylated with (100 mM triethanolamine, pH 8.0, 0.25% acetic anhydride) for 15 minutes on a rocker table and washed 3 × 5 minutes in PBST. Endogenous alkaline phosphatases were inactivated by a 60-minute incubation in PBS at 80°C. The slides were then prehybridized for 60 minutes in hybridization solution (50% formamide, 5X SSC, 5X Denhardt’s, 500 μg/mL herring sperm DNA, 250 μg/mL yeast RNA) at 65°C and hybridized with the digoxigenin-labeled RNA (Roche, Penzberg, Germany) probe (500 ng/mL) overnight at the same temperature. The slides were washed twice in 50% formamide, 2X SSC, 0.1% Tween-20) for 60 minutes and in 0.2X SSC at 65°C for 60 minutes. The slides were then rinsed 3 × 10 minutes in buffer 1 (100 mM maleic acid, pH 7.5, 150 mM NaCl, 0.1% Tween-20), blocked in buffer 2 (buffer 1, 10% sheep serum) for 60 minutes and incubated overnight at 4°C with alkaline phosphatase-coupled anti-DIG antibody (Roche) diluted 1/2000 in buffer 2. The slides were washed 2 × 10 minutes in buffer 1, incubated for 30 minutes in buffer 3 (100 mM Tris, pH 9.5, 100 mM NaCl, 50 mM MgCl_2_, 0.1% Tween-20) and the signal was revealed in filtered buffer 4 (3.5 μL NBT (4-nitroblue tetrazolium chloride) (Roche) 100 mg/mL), 3.5 μL BCIP ((5-bromo-4-chloro-3-indoylphosphate) (Roche) 50 mg/mL) in buffer 3). The slides were washed 3 × 5 minutes in PBST and then postfixed overnight with 4% PFA, washed briefly in PBST and then either washed in water and mounted in Aquatex (Merck) or, when nerves had been retrogradely filled, treated as follows: sections were permeabilized 2 × 10 minutes in PBS, 0.3% Triton X-100, blocked 20 minutes in blocking solution (PBS, 10% fetal calf serum, 0.1% Triton X-100), incubated for two hours in the dark with ExtrAvidin-fluorescein isothiocyanate (FITC, Sigma, Saint-Quentin Fallavier, France) diluted 1/400 in blocking solution, washed 3 × 10 minutes in PBST in the dark and mounted in Mowiol (Calbiochem, Darmstadt, Germany).

### *In situ* hybridization on whole-mounts

The dehydrated desheathed CNS were progressively rehydrated in PBST, incubated for 20 minutes in PBS, 0.3% Triton X-100, rinsed in PBST, permeabilized with PBST, 10 mg/mL Proteinase K for 60 minutes, washed 2 × 5 minutes in PBST, postfixed in 4% PFA for 30 minutes, washed 2 × 5 minutes in PBST, incubated for 10 minutes in 100 mM triethanolamine, pH 8.0, acetylated 2 × 10 minutes with 100 mM triethanolamine, pH 8.0, 0.25% acetic anhydride, washed 2 × 5 minutes in PBST, incubated at 80°C in PBS for 60 minutes, rinsed in PBST, prehybridized for 60 minutes in hybridization buffer (50% formamide, 1.3X SSC, 5 mM EDTA, 50 μg/mL yeast RNA, 2% Tween-20, 0.5% CHAPS) at 65°C and hybridized overnight with the probe (200 ng/mL) at the same temperature. The samples were rinsed twice and washed 2 × 30 minutes in prewarmed hybridization solution at 65°C. Then they were washed for 10 minutes at room temperature in a 1:1 mixture of hybridization solution and buffer 1, 60 minutes in buffer 1, blocked 60 minutes in buffer 1/ 20% FCS, incubated overnight at 4°C with alkaline phosphatase-coupled anti-DIG antibody (Roche) diluted 1/2000 in buffer 1/ 2% FCS, rinsed three times and washed 3 × 60 minutes in buffer 1, then left for two days in buffer 1, incubated 2 × 30 minutes in buffer 3, revealed in the dark with filtered NBT/BCIP (Sigma), washed in the dark 3 x 10 minutes with PBST, postfixed overnight with 4% PFA, rinsed in PBST and kept at 4°C in Tissue-Tek®optimal cutting temperature (O.C.T.) embedding medium (Sakura, Tokyo, Japan). Except for revelation, all steps were performed with agitation.

### Immunofluorescence for detection of mouse Brn3a

Sections were dried, washed for 5 minutes in PBS, permeabilized 2 × 10 minutes in PBS/ 0.3% Triton X-100, blocked for 20 minutes in blocking solution (PBS, 10% FCS, 0.1% Triton X-100), incubated overnight at 4°C with the mouse anti-Brn3a monoclonal antibody (MAB1585) diluted 1/200 in blocking solution, washed 3 × 10 minutes in PBST, incubated for 2 hours with the fluorescent goat anti-mouse Cy3-coupled secondary antibody (115-165-003 Jackson ImmunoResearch, Newmarket, UK), washed 3 × 10 minutes in PBST in the dark and mounted in Mowiol (Calbiochem). The image was converted to gray scale, inverted and superimposed on a Nomarski photomicrograph in Photoshop CS3.

### Cloning of orthologues

Starting with total RNA extracted using RNeasy Lipid Tissue Mini Kit (Qiagen), fragments of newly cloned cDNAs were amplified by PCR and rapid amplification of cDNA ends-PCR (RACE-PCR) as described previously [56]. Orthology was assigned by phylogenetic analysis (see below and Additional file 1: Figure S1). *Ls-VAChT* and *Ls-VGluT* were PCR-amplified using oligonucleotides derived from the GenBank sequences (accession numbers: AF484093 and AB469850, respectively).

### Nerve backfilling

The ganglia of a 20 to 40 mm *Lymnaea stagnalis* specimen were dissected in a 1:1 mixture of physiological medium with Leibovitz's L15 medium (21083–027, Life technologies, Saint Aubin, France) complemented with 50 U/mL pennicilin, 50 μg/mL streptomycin and at the final concentration of 0.3 M glucose, cutting short all nerves except those of interest. The preparation was pinned at the bottom of a sylgard dish where two compartments were delineated with Vaseline, separating the ganglia from the distal part of the nerves to be backfilled. The nerve compartment was emptied, filled with distilled water (dH_2_O), nerves were recut and, after a 10-minute incubation, dH_2_O was replaced by a saturated solution of biocytin (Sigma) in dH_2_O. After 24 hours at room temperature and in the dark, the ganglia were fixed overnight at 4°C in 4% PFA and processed for *in situ* hybridization and biocytin revelation using Extravidin-FITC (see above).

### Phylogenetic analyses

Gene orthologies were assessed using protein sequences aligned with ClustalX2 [57] and the software PhyML [58] with 1,000 bootstrap replicates and the model suggested by ProtTest 2.4 [59]. The closest groups of homeogenes were used as outgroups [60]. Trees were drawn with Dendroscope v3.0.14 [61].

### Ethical approval

Experiments on mouse embryos have been approved by The Research Ethic committee « Charles Darwin », Paris (approval number Ce5/2012/064).

## Abbreviations

CNS: Central nervous system; EDTA: Ethylenediaminetetraacetic acid; FCS: Fetal calf serum; FITC: Fluorescein isthiocyanate; HEPES: 4-(2-hydroxyethyl)-1-piperazineethanesulfonic acid; LYC: Light yellow cells; PBS: Phosphate-buffered saline; PBST: PBS-Tween; PCR: Polymerase chain reaction; PFA: Paraformaldehyde; RACE-PCR: Rapid amplification of cDNA ends-PCR.

## Competing interests

The authors declare that they have no competing interests.

## Authors’ contributions

MN, ZC and HCS isolated genes and performed expression studies; GF and MN performed retrograde filling experiments; SK, LB and EK provided animal tissues from *Aplysia* and *Sepia* and participated in cell identifications; MN and JFB designed the study, analyzed the data and wrote the paper. All authors read and approved the final manuscript.

## Supplementary Material

Additional file 1: Figure S1 Support for orthology assignment of the twelve sequences cloned in this study. Figure S1 shows six phylogenetic trees for genes shared among Bilateria. Figure S2 shows amino acid sequence alignment between the *Aplysia* and *Lymnaea Sensorin* genes. Click here for file
