# Supplementary material for: Ancient origin of somatic and visceral neurons
Source: BMC Biol. 2013 Apr 30;11:53. doi: 10.1186/1741-7007-11-53 (PMC3660236; doi:10.1186/1741-7007-11-53)
Supplement: Additional file 1: Figure S1 — Support for orthology assignment of the twelve sequences cloned in this study. Figure S1 shows six phylogenetic trees for genes shared among Bilateria. Figure S2 shows amino acid sequence alignment between the Aplysia and Lymnaea Sensorin genes. [file 1741-7007-11-53-S1.pdf]

Additional material for

Ancient origin of somatic and visceral neurons

Marc Nomaksteinsky, Stefan Kassabov, Zoubida Chettouh, Henri-Corto Stoeklé, Laure Bonnaud, Gilles Fortin, Eric R. Kandel and Jean-François Brunet

Figures S1a-f and S2

a

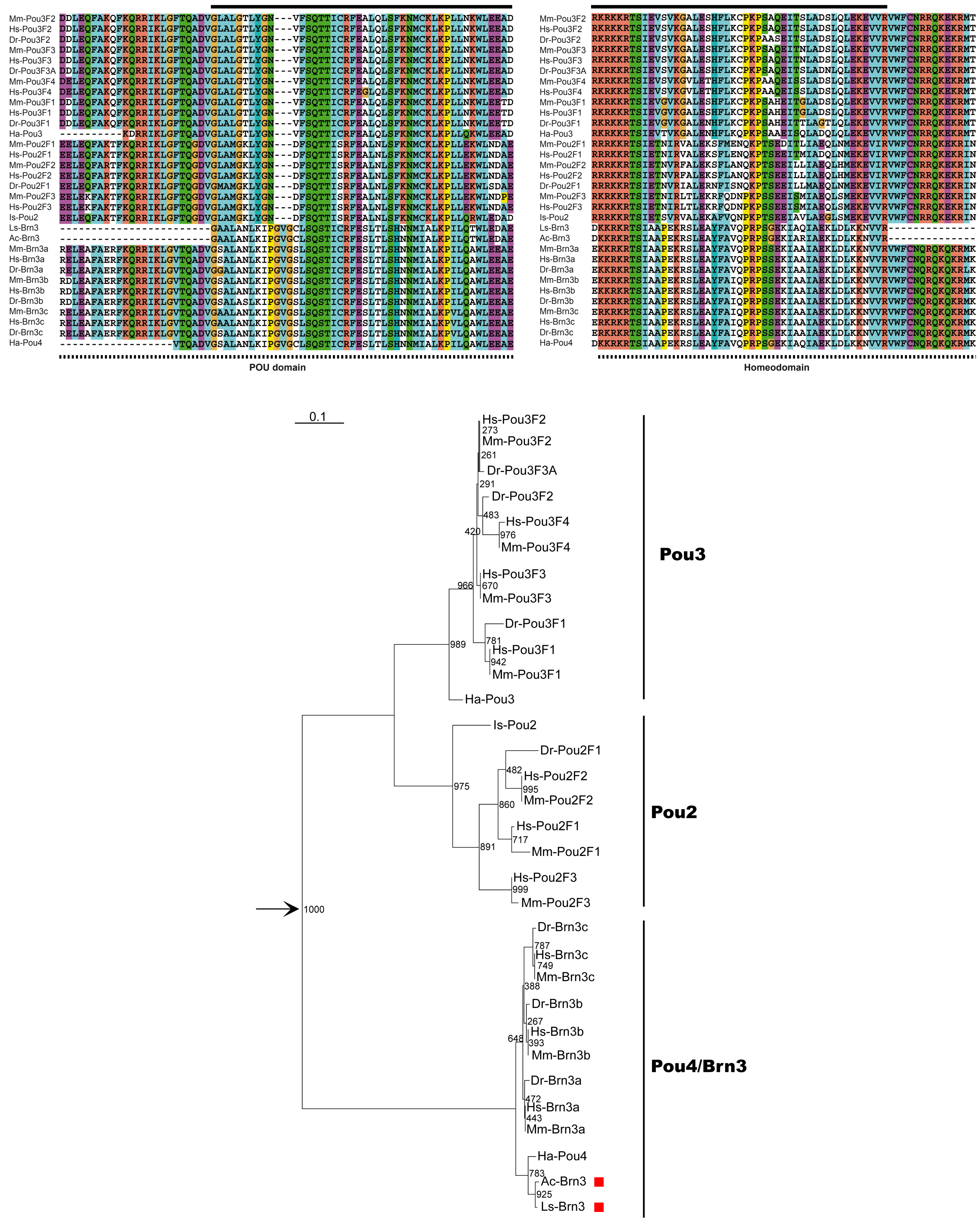

POU domain
Homeodomain
0.1
Pou3
Pou2
Pou4/Brn3
Hs-Pou3F2
Mm-Pou3F2
Dr-Pou3F3A
Dr-Pou3F2
Hs-Pou3F4
Mm-Pou3F4
Hs-Pou3F3
Mm-Pou3F3
Dr-Pou3F1
Hs-Pou3F1
Mm-Pou3F1
Ha-Pou3
Is-Pou2
Dr-Pou2F1
Hs-Pou2F2
Mm-Pou2F2
Hs-Pou2F1
Mm-Pou2F1
Hs-Pou2F3
Mm-Pou2F3
Dr-Brn3c
Hs-Brn3c
Mm-Brn3c
Dr-Brn3b
Hs-Brn3b
Mm-Brn3b
Dr-Brn3a
Hs-Brn3a
Mm-Brn3a
Ha-Pou4
Ac-Brn3
Ls-Brn3
1000

b

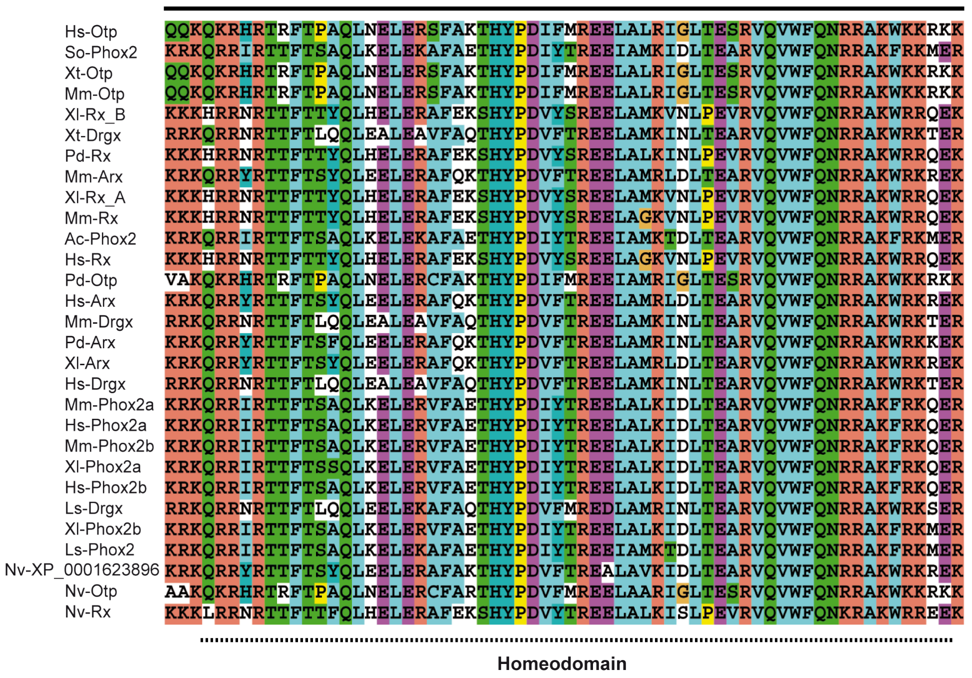

Hs-Otp QQKQKRHRTRFTPAQLNELERSFAKTHYPDIFMREELALRIGLTESRVQVWFQNRRAKWKKRKK
So-Phox2 KRKQRRIRTTFTSAQLKELEKAFAETHYPDIYTREEIAMKIDLTEARVQVWFQNRRAKFRKMER
Xt-Otp QQKQKRHRTRFTPAQLNELERSFAKTHYPDIFMREELALRIGLTESRVQVWFQNRRAKWKKRKK
Mm-Otp QQKQKRHRTRFTPAQLNELERSFAKTHYPDIFMREELALRIGLTESRVQVWFQNRRAKWKKRKK
Xl-Rx_B KKKHRRNRTTFTTYQLHELERAFEKSHYPDVYSREELAMKVNLPEVRVQVWFQNRRAKWRRQEK
Xt-Drgx RRKQRRNRTTFTLQQLEALEAVFAQTHYPDVFTREELAMKINLTEARVQVWFQNRRAKWRKTER
Pd-Rx KKKHRRNRTTFTTYQLHELERAFEKSHYPDVYSREELALKINLPEVRVQVWFQNRRAKWRRQEK
Mm-Arx KRKQRRYRTTFTSYQLEELERAFQKTHYPDVFTREELAMRLDLTEARVQVWFQNRRAKWRKREK
Xl-Rx_A KKKHRRNRTTFTTYQLHELERAFEKSHYPDVYSREELAMKVNLPEVRVQVWFQNRRAKWRRQEK
Mm-Rx KKKHRRNRTTFTTYQLHELERAFEKSHYPDVYSREELAGKVNLPEVRVQVWFQNRRAKWRRQEK
Ac-Phox2 KRKQRRIRTTFTSAQLKELEKAFAETHYPDIYTREEIAMKTDLTEARVQVWFQNRRAKFRKMER
Hs-Rx KKKHRRNRTTFTTYQLHELERAFEKSHYPDVYSREELAGKVNLPEVRVQVWFQNRRAKWRRQEK
Pd-Otp VAKQKRHRTRFTPAQLNELERCFAKTHYPDIFMREEIAMRIGLTESRVQVWFQNRRAKWKKRKK
Hs-Arx KRKQRRYRTTFTSYQLEELERAFQKTHYPDVFTREELAMRLDLTEARVQVWFQNRRAKWRKREK
Mm-Drgx RRKQRRNRTTFTLQQLEALEAVFAQTHYPDVFTREELAMKINLTEARVQVWFQNRRAKWRKTER
Pd-Arx KRKQRRYRTTFTSFQLEELERAFQKTHYPDVFTREELAMRINLTEARVQVWFQNRRAKWRKKEK
Xl-Arx KRKQRRYRTTFTSYQLEELERAFQKTHYPDVFTREELAMRLDLTEARVQVWFQNRRAKWRKREK
Hs-Drgx RRKQRRNRTTFTLQQLEALEAVFAQTHYPDVFTREELAMKINLTEARVQVWFQNRRAKWRKTER
Mm-Phox2a KRKQRRIRTTFTSAQLKELERVFAETHYPDIYTREELALKIDLTEARVQVWFQNRRAKFRKQER
Hs-Phox2a KRKQRRIRTTFTSAQLKELERVFAETHYPDIYTREELALKIDLTEARVQVWFQNRRAKFRKQER
Mm-Phox2b KRKQRRIRTTFTSAQLKELERVFAETHYPDIYTREELALKIDLTEARVQVWFQNRRAKFRKQER
Xl-Phox2a KRKQRRIRTTFTSSQLKELERVFAETHYPDIYTREELALKIDLTEARVQVWFQNRRAKFRKQER
Hs-Phox2b KRKQRRIRTTFTSAQLKELERVFAETHYPDIYTREELALKIDLTEARVQVWFQNRRAKFRKQER
Ls-Drgx RRKQRRNRTTFTLQQLEELEKAFAQTHYPDVFMREDLAMRINLTEARVQVWFQNRRAKWRKSER
Xl-Phox2b KRKQRRIRTTFTSAQLKELERVFAETHYPDIYTREELALKIDLTEARVQVWFQNRRAKFRKMER
Ls-Phox2 KRKQRRIRTTFTSAQLKELEKAFAETHYPDIYTREEIAMKTDLTEARVQVWFQNRRAKFRKMER
Nv-XP_0001623896 KRKQRRYRTTFTSYQLEELERAFAKTHYPDVFTREALAVKIDLTEARVQVWFQNRRAKWRKREK
Nv-Otp AAKQKRHRTRFTPAQLNELERCFARTHYPDVFMREELAARIGLTESRVQVWFQNRRAKWKKRKK
Nv-Rx KKKLRRNRTTFTTFQLHELERAFEKSHYPDVYTREELALKISLPEVRVQVWFQNKRAKWRREEK
Homeodomain

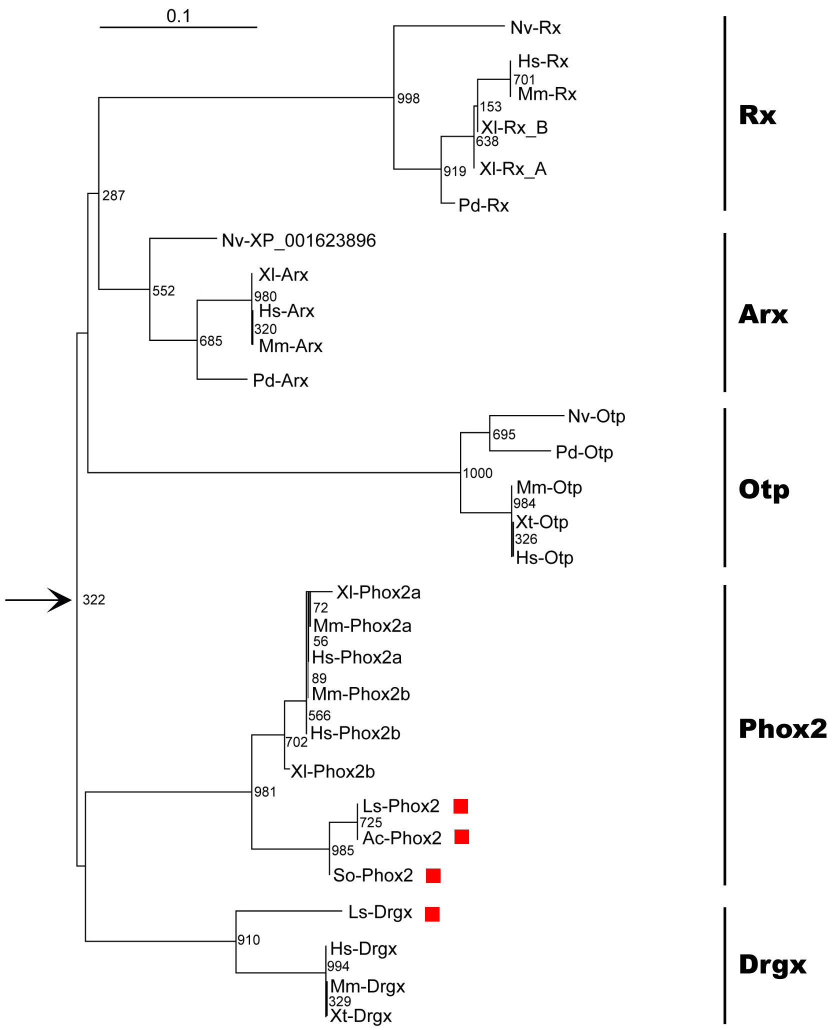

0.1
Nv-Rx
Hs-Rx
701
Mm-Rx
153
Xl-Rx_B
638
998
919
Xl-Rx_A
Pd-Rx
Rx
287
Nv-XP_001623896
Xl-Arx
980
Hs-Arx
552
320
685
Mm-Arx
Pd-Arx
Arx
Nv-Otp
695
Pd-Otp
1000
Mm-Otp
984
Xt-Otp
326
Hs-Otp
Otp
Xl-Phox2a
72
Mm-Phox2a
56
Hs-Phox2a
89
Mm-Phox2b
566
Hs-Phox2b
702
Xl-Phox2b
981
322
Ls-Phox2
725
Ac-Phox2
985
So-Phox2
Phox2
Ls-Drgx
910
Hs-Drgx
994
Mm-Drgx
329
Xt-Drgx
Drgx

c

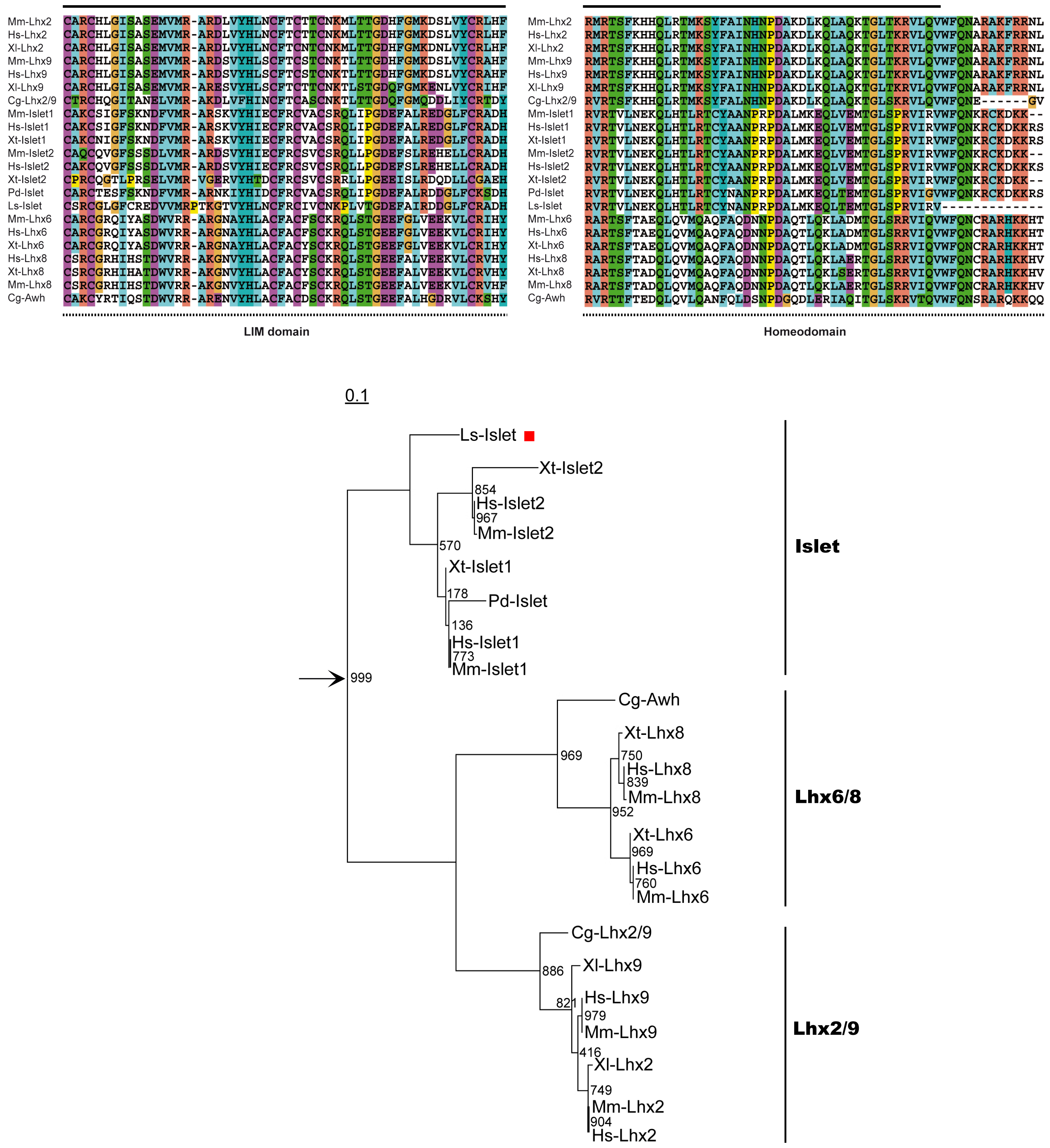
Mm-Lhx2
Hs-Lhx2
Xl-Lhx2
Mm-Lhx9
Hs-Lhx9
Xl-Lhx9
Cg-Lhx2/9
Mm-Islet1
Hs-Islet1
Xt-Islet1
Mm-Islet2
Hs-Islet2
Xt-Islet2
Pd-Islet
Ls-Islet
Mm-Lhx6
Hs-Lhx6
Xt-Lhx6
Hs-Lhx8
Xt-Lhx8
Mm-Lhx8
Cg-Awh
LIM domain
Homeodomain
0.1
Ls-Islet
Xt-Islet2
854
Hs-Islet2
967
Mm-Islet2
570
Xt-Islet1
178
Pd-Islet
136
Hs-Islet1
773
Mm-Islet1
999
Islet
Cg-Awh
Xt-Lhx8
969
750
Hs-Lhx8
839
Mm-Lhx8
952
Xt-Lhx6
969
Hs-Lhx6
760
Mm-Lhx6
Lhx6/8
Cg-Lhx2/9
Xl-Lhx9
886
821
Hs-Lhx9
979
Mm-Lhx9
416
Xl-Lhx2
749
Mm-Lhx2
904
Hs-Lhx2
Lhx2/9

d

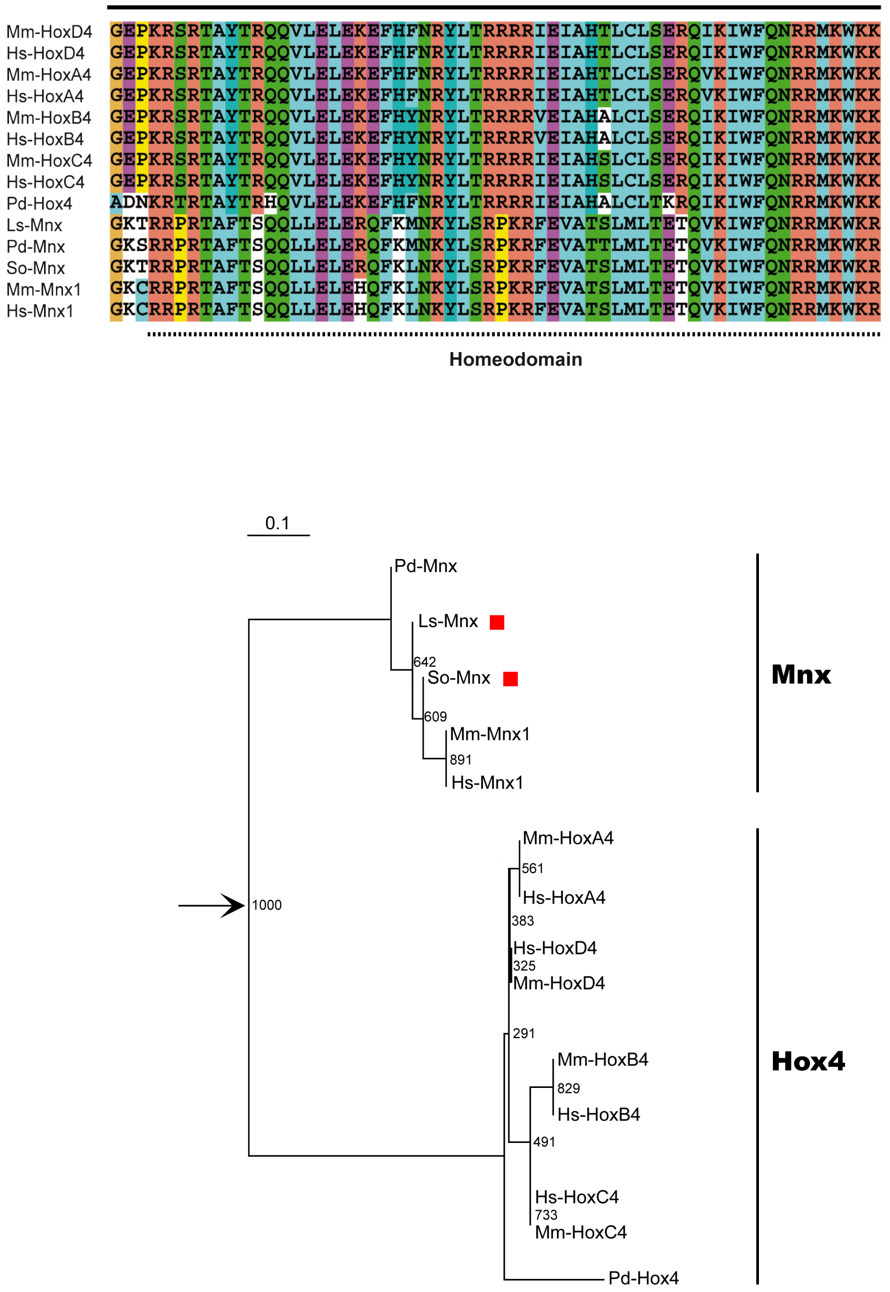

Mm-HoxD4 GEPKRSRTAYTRQQVLELEKEFHFNRYLTRRRRIEIAHTLCLSERQIKIWFQNRRMKWKK
Hs-HoxD4 GEPKRSRTAYTRQQVLELEKEFHFNRYLTRRRRIEIAHTLCLSERQIKIWFQNRRMKWKK
Mm-HoxA4 GEPKRSRTAYTRQQVLELEKEFHFNRYLTRRRRIEIAHTLCLSERQVKIWFQNRRMKWKK
Hs-HoxA4 GEPKRSRTAYTRQQVLELEKEFHFNRYLTRRRRIEIAHTLCLSERQVKIWFQNRRMKWKK
Mm-HoxB4 GEPKRSRTAYTRQQVLELEKEFHYNRYLTRRRRVEIAHALCLSERQIKIWFQNRRMKWKK
Hs-HoxB4 GEPKRSRTAYTRQQVLELEKEFHYNRYLTRRRRVEIAHALCLSERQIKIWFQNRRMKWKK
Mm-HoxC4 GEPKRSRTAYTRQQVLELEKEFHYNRYLTRRRRIEIAHSLCLSERQIKIWFQNRRMKWKK
Hs-HoxC4 GEPKRSRTAYTRQQVLELEKEFHYNRYLTRRRRIEIAHSLCLSERQIKIWFQNRRMKWKK
Pd-Hox4 ADNKRTRTAYTRHQVLELEKEFHFNRYLTRRRRIEIAHALCLTKRQIKIWFQNRRMKWKK
Ls-Mnx GKTRRPRTAFTSQQLLELERQFKMNKYLSRPKRFEVATSLMLTETQVKIWFQNRRMKWKR
Pd-Mnx GKSRRPRTAFTSQQLLELERQFKMNKYLSRPKRFEVATTLMLTETQVKIWFQNRRMKWKR
So-Mnx GKTRRPRTAFTSQQLLELERQFKLNKYLSRPKRFEVATSLMLTETQVKIWFQNRRMKWKR
Mm-Mnx1 GKCRRPRTAFTSQQLLELEHQFKLNKYLSRPKRFEVATSLMLTETQVKIWFQNRRMKWKR
Hs-Mnx1 GKCRRPRTAFTSQQLLELEHQFKLNKYLSRPKRFEVATSLMLTETQVKIWFQNRRMKWKR
Homeodomain
0.1
Pd-Mnx
Ls-Mnx
642
So-Mnx
609
Mm-Mnx1
891
Hs-Mnx1
Mnx
Mm-HoxA4
561
Hs-HoxA4
1000
383
Hs-HoxD4
325
Mm-HoxD4
291
Mm-HoxB4
829
Hs-HoxB4
491
Hs-HoxC4
733
Mm-HoxC4
Pd-Hox4
Hox4

e

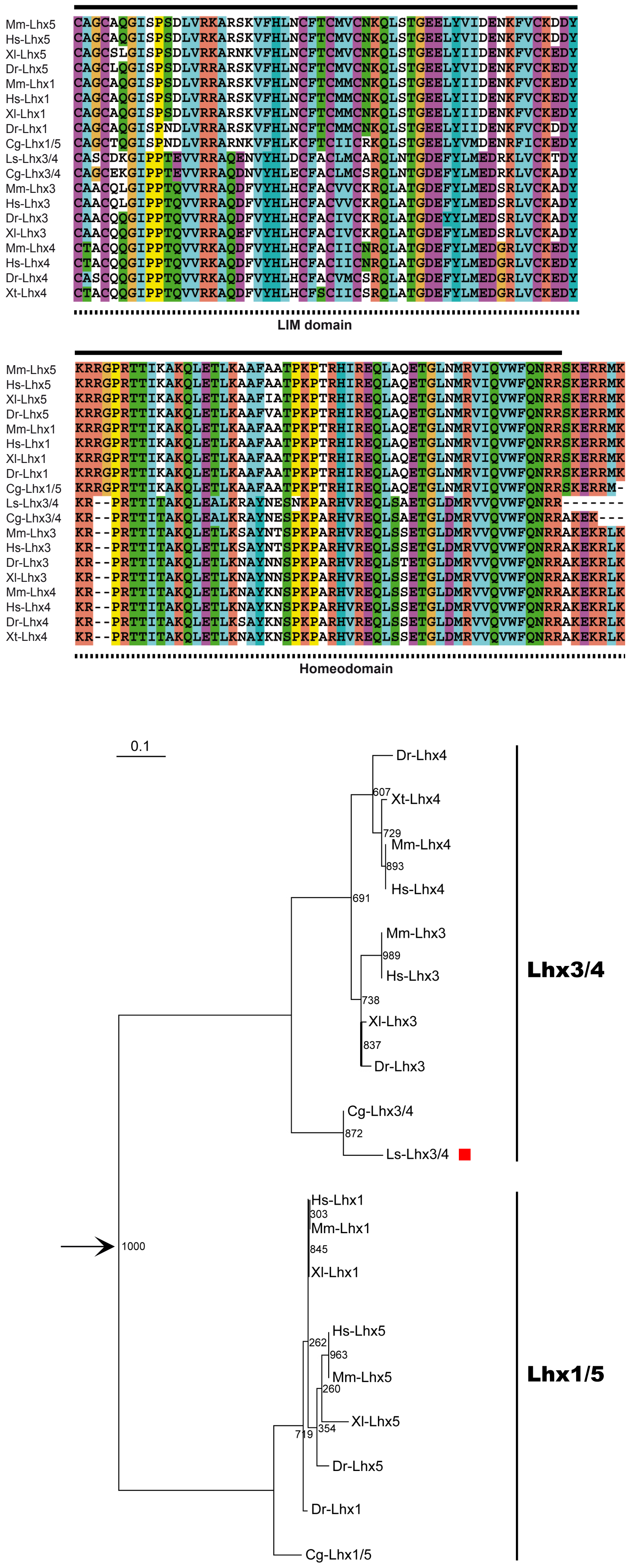

Mm-Lhx5
Hs-Lhx5
Xl-Lhx5
Dr-Lhx5
Mm-Lhx1
Hs-Lhx1
Xl-Lhx1
Dr-Lhx1
Cg-Lhx1/5
Ls-Lhx3/4
Cg-Lhx3/4
Mm-Lhx3
Hs-Lhx3
Dr-Lhx3
Xl-Lhx3
Mm-Lhx4
Hs-Lhx4
Dr-Lhx4
Xt-Lhx4
LIM domain
Homeodomain
0.1
Dr-Lhx4
607
Xt-Lhx4
729
Mm-Lhx4
893
Hs-Lhx4
691
Mm-Lhx3
989
Hs-Lhx3
738
Xl-Lhx3
837
Dr-Lhx3
Cg-Lhx3/4
872
Ls-Lhx3/4
Lhx3/4
Hs-Lhx1
303
Mm-Lhx1
845
1000
Xl-Lhx1
Hs-Lhx5
262
963
Mm-Lhx5
260
Xl-Lhx5
354
719
Dr-Lhx5
Dr-Lhx1
Cg-Lhx1/5
Lhx1/5

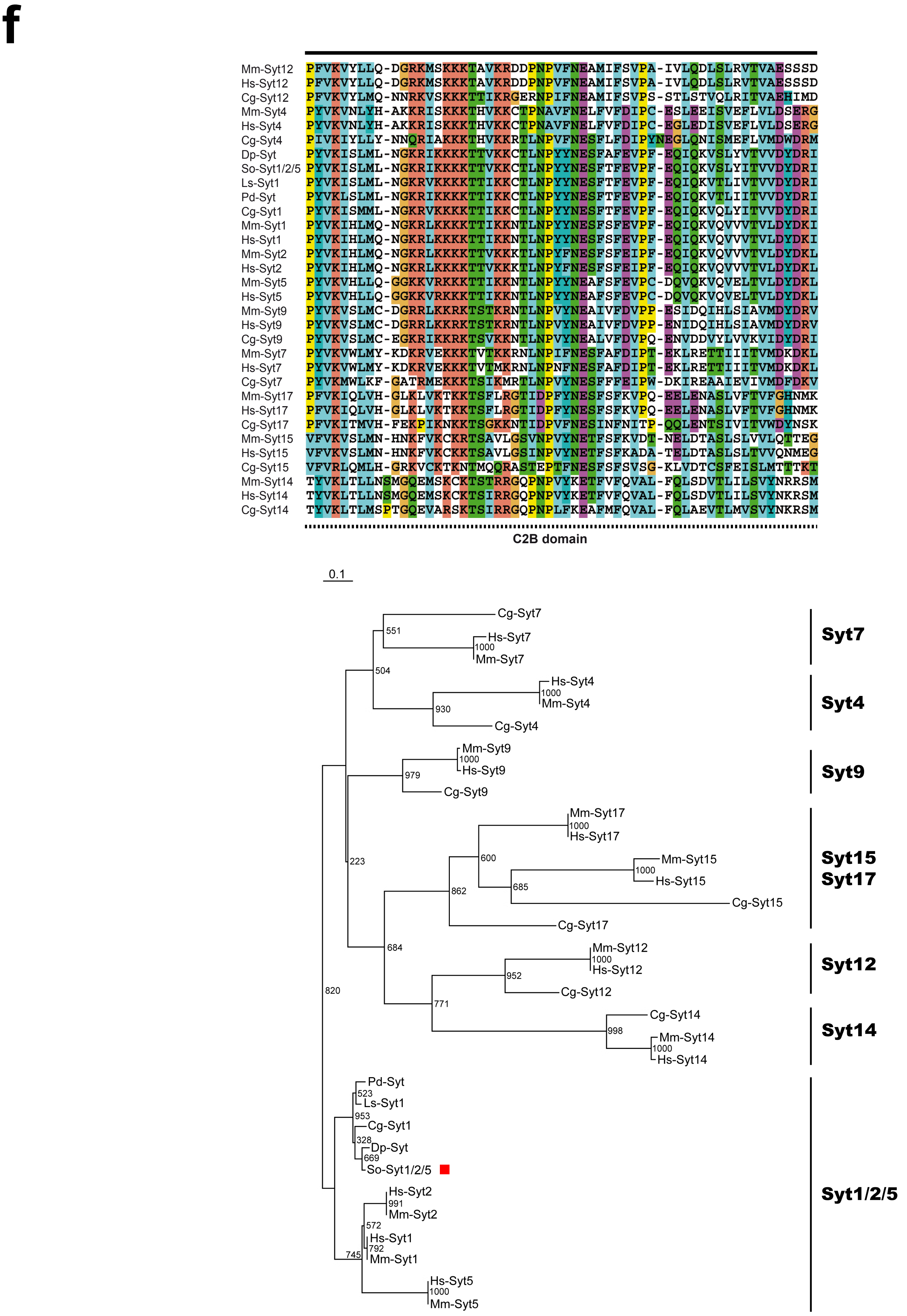
f
Mm-Syt12 PFVKVYLLQ-DGRKMSKKKTAVKRDDPNPVFNEAMIFSVPA-IVLQDLSLRVTVAESSSD
Hs-Syt12 PFVKVYLLQ-DGRKMSKKKTAVKRDDPNPVFNEAMIFSVPA-IVLQDLSLRVTVAESSSD
Cg-Syt12 PFVKVYLMQ-NNRKVSKKKTTIKRGERNPIFNEAMIFSVPS-STLSTVQLRITVAEHIMD
Mm-Syt4 PYVKVNLYH-AKKRISKKKTHVKKCTPNAVFNELFVFDIPC-ESLEEISVEFLVLDSERG
Hs-Syt4 PYVKVNLYH-AKKRISKKKTHVKKCTPNAVFNELFVFDIPC-EGLEDISVEFLVLDSERG
Cg-Syt4 PIVKIYLLY-NNQRIAKKKTHVKKRTLNPVFNESFLFDIPYNEGLQNISMEFLVMDWDRM
Dp-Syt PYVKISLML-NGKRIKKKKTTVKKCTLNPYYNESFAFEVPF-EQIQKVSLYVTVVDYDRI
So-Syt1/2/5 PYVKISLML-NGKRIKKKKTTVKKCTLNPYYNESFTFEVPF-EQIQKVSLYITVVDYDRI
Ls-Syt1 PYVKISLML-NGKRVKKKKTTIKKCTLNPYYNESFTFEVPF-EQIQKVTLIVTVVDYDRI
Pd-Syt PYVKLSLML-NGKRIKKKKTTIKKCTLNPYYNESFTFEVPF-EQIQKVTLIITVVDYDRI
Cg-Syt1 PYVKISMML-NGKRVKKKKTTIKKCTLNPYYNESFTFEVPF-EQIQKVQLYITVVDYDRI
Mm-Syt1 PYVKIHLMQ-NGKRLKKKKTTIKKNTLNPYYNESFSFEVPF-EQIQKVQVVVTVLDYDKI
Hs-Syt1 PYVKIHLMQ-NGKRLKKKKTTIKKNTLNPYYNESFSFEVPF-EQIQKVQVVVTVLDYDKI
Mm-Syt2 PYVKIHLMQ-NGKRLKKKKTTVKKKTLNPYFNESFSFEIPF-EQIQKVQVVVTVLDYDKL
Hs-Syt2 PYVKIHLMQ-NGKRLKKKKTTVKKKTLNPYFNESFSFEIPF-EQIQKVQVVVTVLDYDKL
Mm-Syt5 PYVKVHLLQ-GGKKVRKKKTTIKKNTLNPYYNEAFSFEVPC-DQVQKVQVELTVLDYDKL
Hs-Syt5 PYVKVHLLQ-GGKKVRKKKTTIKKNTLNPYYNEAFSFEVPC-DQVQKVQVELTVLDYDKL
Mm-Syt9 PYVKVSLMC-DGRRLKKRKTSTKRNTLNPVYNEAIVFDVPP-ESIDQIHLSIAVMDYDRV
Hs-Syt9 PYVKVSLMC-DGRRLKKRKTSTKRNTLNPVYNEAIVFDVPP-ENIDQIHLSIAVMDYDRV
Cg-Syt9 PYVKVSLMC-EGKRIKKRKTSVKKNTLNPVYNEALVFDVPQ-ENVDDVYLVVKVIDYDRI
Mm-Syt7 PYVKVWLMY-KDKRVEKKKTVTKKRNLNPIFNESFAFDIPT-EKLRETTIIITVMDKDKL
Hs-Syt7 PYVKVWLMY-KDKRVEKKKTVTMKRNLNPNFNESFAFDIPT-EKLRETTIIITVMDKDKL
Cg-Syt7 PYVKMWLKF-GATRMEKKKTSIKMRTLNPVYNESFFFEIPW-DKIREAAIEVIVMDFDKV
Mm-Syt17 PFVKIQLVH-GLKLVKTKKTSFLRGTIDPFYNESFSFKVPQ-EELENASLVFTVFGHNMK
Hs-Syt17 PFVKIQLVH-GLKLVKTKKTSFLRGTIDPFYNESFSFKVPQ-EELENASLVFTVFGHNMK
Cg-Syt17 PFVKITMVH-FEKPIKNKKTSGKKNTIDPVFNESINFNITP-QQLENTSIVITVWDYNSK
Mm-Syt15 VFVKVSLMN-HNKFVKCKRTSAVLGSVNPVYNETFSFKVDT-NELDTASLSLVVLQTTEG
Hs-Syt15 VFVKVSLMN-HNKFVKCKKTSAVLGSINPVYNETFSFKADA-TELDTASLSLTVVQNMEG
Cg-Syt15 VFVRLQMLH-GRKVCKTKNTMQQRASTEPTFNESFSFSVSG-KLVDTCSFEISLMTTTKT
Mm-Syt14 TYVKLTLLNSMGQEMSKCKTSTRRGQPNPVYKETFVFQVAL-FQLSDVTLILSVYNRRSM
Hs-Syt14 TYVKLTLLNSMGQEMSKCKTSIRRGQPNPVYKETFVFQVAL-FQLSDVTLILSVYNKRSM
Cg-Syt14 TYVKLTLMSPTGQEVARSKTSIRRGQPNPLFKEAFMFQVAL-FQLAEVTLMVSVYNKRSM
C2B domain
0.1
Cg-Syt7
551
Hs-Syt7
1000
Mm-Syt7
504
Hs-Syt4
1000
Mm-Syt4
930
Cg-Syt4
Mm-Syt9
1000
Hs-Syt9
979
Cg-Syt9
Mm-Syt17
1000
Hs-Syt17
223
600
Mm-Syt15
1000
Hs-Syt15
685
862
Cg-Syt15
Cg-Syt17
684
Mm-Syt12
1000
Hs-Syt12
952
Cg-Syt12
820
771
Cg-Syt14
998
Mm-Syt14
1000
Hs-Syt14
Pd-Syt
523
Ls-Syt1
953
Cg-Syt1
328
Dp-Syt
669
So-Syt1/2/5
Hs-Syt2
991
Mm-Syt2
572
Hs-Syt1
792
745
Mm-Syt1
Hs-Syt5
1000
Mm-Syt5
Syt7
Syt4
Syt9
Syt15
Syt17
Syt12
Syt14
Syt1/2/5

Figure S1. Sequence alignments and Maximum Likelihood (ML) phylogenetic trees (1000 bootstrap replicates) for *Aplysia* and *Lymnaea Brn3* (**a**), *Aplysia, Lymnaea*, *Sepia Phox2* and *Lymnaea Drgx* (**b**), *Lymnaea Islet* (**c**), *Lymnaea* and *Sepia Mnx* (**d**), *Lymnaea Lhx3/4 (***e***)* and *Sepia Synaptotagmin 1/2/5* (**f**).
The amino acids included in each ML analysis are indicated by an horizontal bar above the alignment. Scale is substitutions per site. The genes cloned in this study are indicated by a red square. The root of the tree is indicated by an arrow.
Newly cloned sequences from *Lymnaea*, *Aplysia* and *Sepia* have been deposited in Genbank under the following accession numbers: Sepia *Synaptotagmin1/2/5*, JX983553; Lymnaea *Brn3*, JX971965; Aplysia *Brn3*, JX971966; Lymnaea *Sensorin-A*, JX971967; Lymnaea *Phox2*, JX971968; Aplysia *Phox2*, JX971969; Sepia *Phox2*, JX971970; Lymnaea *Islet*, JX971971; Lymnaea *Lhx3/4*, JX971972; Lymnaea *Mnx*, JX971973; Sepia *Mnx*, JX971974; Lymnaea *Drgx*, JX971975.
Species abbreviations*: Ac, Aplysia californica; Cg, Crassostrea gigas; Dp, Doryteuthis pealeii; Dr, Danio rerio; Hs, Homo sapiens; Is, Ixodes scapularis; Ls, Lymnaea stagnalis; Mm, Mus musculus; Nv, Nematostella vectensis; Pd, Platynereis dumerilii; So, Sepia officinalis; Xl, Xenopus laevis; Xt, Xenopus tropicalis.*

putative cleavage site

peptide B

peptide A

```
Ac Sensorin A  MPSRAATSPLNVQMMVVLCIVCLALQAVAANATRSKNNVPRRFPRARYRVGYMFGKRSSSETYST 65
Ls Sensorin A                MSPTNMSSGLVPVLVLTVVACLMLTDNVSASARYRVGYMFGKRG-IESPST 50
                                                             ************   *  **

Ac Sensorin A  NLINLLSRQLVSQEELRAILEKQPILLDEVVKILDRNDDGYITVADLL 113
Ls Sensorin A  SFIDTISREMKTKQEVESLILKNPEVLSEVLTILDKNDDGYITVSDVL  98
                 *   **      *      * *  * **  *** ******** * *
```

Figure S2. Alignment of the Sensorin amino acid sequences of *Aplysia californica* (Ac) and *Lymnaea stagnalis* (Ls). A star underlies conserved residues and an hyphen represents a gap.
